# Unmanipulated haploidentical stem cell transplantation in adults with acute lymphoblastic leukemia: a study on behalf of the Acute Leukemia Working Party of the EBMT

**DOI:** 10.1186/s13045-017-0480-5

**Published:** 2017-05-30

**Authors:** Nicole Santoro, Annalisa Ruggeri, Myriam Labopin, Andrea Bacigalupo, Fabio Ciceri, Zafer Gülbaş, He Huang, Boris Afanasyev, William Arcese, Depei Wu, Yener Koc, Johanna Tischer, Stella Santarone, Sebastian Giebel, Mohamad Mohty, Arnon Nagler

**Affiliations:** 1 Department of Hematology and Cell Therapy, Hôpital Saint-Antoine, 184 Rue du Faubourg Saint Antoine, 75012 Paris, France; 2 Department of Medicine, Division of Hematology and Clinical Immunology, University of Perugia, Perugia, Italy; 3 ALWP office, Hôpital Saint-Antoine, Paris, France; 4 Department of Hematology II, Ospedale San Martino, Genova, Italy; 5 Università cattolica del Sacro Cuore, Roma, Italy; 6 Hematology and Bone Marrow Transplantation Unit, IRCCS San Raffaele Scientific Institute, Milan, Italy; 7 Hematology Department, Anadolu Medical Center Hospital, Kocaeli, Turkey; 8 Bone Marrow Transplantation Center, The First Affiliated Hospital, School of Medicine, Zhejiang University School of Medicine, Hangzhou, Zhejiang China; 9 Hematology and Transplantology, Ratsa Gorbacheva Memorial Children’s Institute, Saint Petersburg State Medical Pavlov University, St. Petersburg, Russian Federation; 10 Stem Cell Transplant Unit, Department of Hematology, Tor Vergata “University Hospital”, Rome, Italy; 11 Department of Hematology, The First Affiliated Hospital of Soochow University, Suzhou, Jiangsu China; 12 Stem Cell Transplant Unit, Medical Park Hospitals, Antalya, Turkey; 13 Department of Internal Medicine III, Hematopoietic Stem Cell Transplantation, Ludwig-Maximilians-University Hospital of Munich-Grosshadern, Munich, Germany; 14 Department of Hematology and Trasfusional Medicine, Lund University, Ospedale Civile, Pescara, Italy; 15 Department of Bone Marrow Transplantation and Onco-Hematology, Comprehensive Cancer Center M. Sklodowska-Curie Memorial Institute, Gliwice Branch, Gliwice, Poland; 16 Department of Hematology and Bone Marrow Transplantation, Chaim Sheba Medical Center, Tel Aviv, Israel

**Keywords:** Non-TCD haploidentical, Allogenic stem cell transplantation, Acute lymphoblastic leukemia, Adults

## Abstract

**Background:**

Allogenic hematopoietic stem cell transplantation (allo-SCT) is the most effective post-remission treatment for adults with high-risk acute lymphoblastic leukemia (ALL). The aim of the study was to analyze results of unmanipulated haploidentical allo-SCT (haplo-SCT) for adults with ALL and to identify prognostic factors.

**Methods:**

We performed a retrospective analysis on 208 adults transplanted in EBMT centers from 2007 to 2014.

**Results:**

Median age at haplo-SCT was 32 years and median follow-up, 31 months. Forty-four percent of the patients were in first complete remission (CR1). Stem cell source was the bone marrow (BM) for 43% and peripheral blood (PB) for 57% of patients. Myeloablative conditioning (MAC) was used for 66% and reduced intensity regimen (RIC) for 34% of patients. GVHD prophylaxis was based on post-transplant cyclophosphamide (PT-Cy) for 118 (57%) or on anti-thymocyte-globulin (ATG) for 90 (43%) plus standard prophylaxis. One hundred eighty-four (92%) patients achieved engraftment. Cumulative incidence (CI) of grade II–IV acute-graft-versus-host-disease (GVHD) was 31%, grade III–IV 11%, and chronic GVHD 29%. Non-relapse mortality (NRM) and relapse-incidence (RI) were 32 and 37%, respectively. Overall survival (OS), leukemia-free survival (LFS), and GVHD-free, relapse-free-survival (GRFS) at 3 years were 33, 31, and 26%. For patients in CR1, OS, LFS, and GRFS were 52, 47, and 40%, respectively. Disease status was the main factor associated with transplant outcomes. Use of BM was independently associated with improvement in NRM, acute GVHD, GRFS, LFS, and OS.

**Conclusions:**

Unmanipulated haplo-SCT may be considered a valid option for adult patients with high-risk ALL lacking HLA identical donor preferably in early disease status.

**Electronic supplementary material:**

The online version of this article (doi:10.1186/s13045-017-0480-5) contains supplementary material, which is available to authorized users.

## Background

Acute lymphoblastic leukemia (ALL) has a high frequency in children and young adults [1–3] and is rare in adults. Childhood ALL is associated with cure rates higher than 80%, while in adult patients’ cure rates range between 20 and 40% with high incidence of relapse and dismal prognosis [4, 5]. Some of the differences between childhood and adult ALL are related to the biology of the disease, treatment, and patient-related factors. Adverse genetic features in adults predispose to chemotherapy resistance and disease relapse after an initial achievement of complete remission (CR). Other factors are the high incidence of comorbidities and treatment-related side effects that may result in the administration of lower cumulative doses of chemotherapy [4].

Approximately 80% of the patients with adult ALL achieve first CR (CR1); however, the major barrier to long-term survival is the disease recurrence which is over 60% with a median overall survival (OS) of <10 months [6].

Besides some controversy on indication and timing, allogenic hematopoietic stem cell transplantation (allo-SCT) is the standard of care for high-risk patients in CR1 and all patients who experience relapse [7–12].

The most suitable donor for transplantation is an HLA-matched sibling or fully matched (10/10 HLA matched) unrelated donor (MUD). However, for patients who lack HLA matched sibling or MUD, allo-SCT from un haploidentical or cord blood donor, is a possible option [13, 14].

Haploidentical hematopoietic stem cell transplantation (haplo-SCT) is increasingly used [15] since it allows almost all patients in need for an allo-SCT to undergo allo-SCT. Haploidentical donors (siblings, children, parents, and extended relatives) are virtually available for all patients, and the access to further stem cell donations or donor lymphocyte infusions (DLIs) or other types of adoptive cellular therapies is easily available.

In the last decade, unmanipulated grafts without T cell depletion (TCD) have been used more frequently in the haplo-setting with the use of anti-thymocyte globulins (ATG) or post-transplant cyclophosphamide (PT-Cy) as GVHD prophylaxis with encouraging results [16–21]. Furthermore, the optimization of conditioning regimens with the development of reduced intensity conditioning (RIC) including for haplo-SCT, have further extended the use of haplo-SCT to older patients and those with significant pre-transplant comorbidities [22].

Several single-center- and registry-based studies showed comparable outcomes between haplo-SCT and mismatched (9/10 HLA compatibility) unrelated donor or cord blood transplants in patients with acute leukemias [23–25]. However, few studies, so far, analyzed the results of haplo-SCT in adult ALL.

In a recent report by Srour et al. [26], outcomes of 109 adults with ALL receiving haplo-SCT with PT-Cy as GVHD prophylaxis were reported with encouraging results. We conducted a registry-based study of adults with ALL transplanted in EBMT centers using unmanipulated haplo-SCT with PT-Cy or ATG as GVHD prophylaxis and MAC or RIC as conditioning regimens.

## Methods

### Study design

This is a retrospective registry-based analysis on behalf of the ALWP of EBMT on haplo-SCT, in adult patients with ALL, performed between January 2007 and December 2014.

The EBMT is a voluntary working group of more than 500 transplant centers that are required to report all consecutive stem cell transplantations and follow-ups once a year. Audits are routinely performed to determine the accuracy of the data.

Patients included into the study, fulfilled all of the following criteria: age ≥18 years; de novo ALL; first allo-SCT, host–donor number of HLA mismatches ≥2; peripheral blood (PB) or bone marrow (BM) grafts and no ex vivo T cell depletion.

Minimal residual disease (MRD) was defined as any evidence of detectable disease by cytogenetics, flow-cytometry, and/or polymerase chain reaction (PCR) for patients in morphologic remission at transplant.

MAC was defined as a regimen containing either total body irradiation (TBI) with a dose greater than 6 Gy, a total dose of oral busulfan (Bu) greater than 8 mg/kg, or a total dose of intravenous Bu greater than 6.4 mg/kg or melphalan at doses >140 mg/m^2^. In addition, regimens containing two alkylating agents were considered as MAC. All other regimens were defined as RIC [27].

### Statistical analysis

The primary endpoint was leukemia-free survival (LFS). Secondary endpoints were overall survival (OS), refined graft-versus-host-free, relapse-free survival (GRFS), neutrophil engraftment, acute (a)GVHD and chronic (c)GVHD, relapse incidence (RI), and non-relapse mortality (NRM).

LFS was defined as the interval from haplo-SCT to either relapse or death in remission. OS was defined as the time to death from all causes. GRFS events have been defined as grade 3–4 acute GVHD, severe chronic GVHD, disease relapse, or death from any cause after SCT [28]. Engraftment was defined as the first of three consecutive days with an absolute neutrophil count >0.5 × 10^9^/l. aGVHD was graded according to the modified Glucksberg criteria [29] and cGVHD according to the revised Seattle criteria [30].

Cumulative incidence (CI) of relapse and NRM was calculated from the date of transplant to the date of relapse or death in remission, respectively, with the other event being the competing risk. For studying GVHD, both relapse and death were considered as competing events.

Univariate comparisons of time-dependent endpoints were done using the log-rank test for OS and LFS and GRFS and the Gray’s test for cumulative incidence functions.

A multivariate analysis was performed using Cox proportional hazards model. Variables were included in the multivariate model if they were conceptually important or if they approached or attained statistical significance by univariate analysis. All tests are two-sided. The type I error rate was fixed at 0.05 for determination of factors associated with time to event.

In order to test for a center effect, we introduced a random effect or frailty for each center into the model [31, 32].

Statistical analyses were performed with the SPSS 22 (SPSS Inc./IBM, Armonk, NY, USA) and R 3.2.3 (R Development Core Team, Vienna, Austria) software packages.

## Results

### Patients, disease, and transplant characteristics

A total of 208 patients transplanted in 69 EBMT centers were analyzed, and a median of eight haplo-SCT for each center was reported. No center effect was found using the frailty model (*p* = 0.30). Patient, disease, and transplant characteristics are listed in Table 1.

**Table 1:** Patients, disease, and transplant characteristics

Variables		
Follow-up (months)	Median (range)	31 (2–79)
Patient age (years)	Median (range)	32 (18–76)
Year of Tx	Median (range)	2012 (2007–2014)
Patient sex	Female	80 (39%)
	Male	127 (61%)
Karnofsky at Tx	≥90%	128 (69%)
Previous autologous Tx	Yes	14 (7%)
Disease status at Tx	CR1	91 (44%)
	CR2+	58 (28%)
	Advanced disease	59 (28%)
Immunophenotype	B ALL	100 (66%)
	T ALL	51 (34%)
CNS involvement	Yes	10 (10%)
Status of MRD at Tx	Positive	45 (49%)
	Negative	46 (51%)
Cytogenetics	Ph-positive	46 (32%)
	Ph-negative	96 (68%)
	*Normal karyotype*	*37*
	*Abnormal karyotype*	*32*
	*t*(*4*;*11*)	*4*
	*t*(*1*;*19*)	*5*
	*t*(*12*;*21*)	*2*
Donor age	*Median* (*range*)	39 (12–74)
F donor/M recipient	Yes	63 (30%)
Stem cell source	BM	89 (43%)
	PB	119 (57%)
CMV D/R	Neg to neg	24 (12%)
	Pos to neg	20 (10%)
	Neg to pos	30 (15%)
	Pos to pos	127 (63%)
Donor kinship	Parents	49 (37%)
	Sibling	51 (38%)
	Child	25 (19%)
	Others relatives	8 (6%)
Conditioning regimen	MAC	*137* (*66%*)
	TBI-based (8–12 Gy)	63
	*Cy TBI*	*29*
	*Flu TBI*	*31*
	*Other TBI*	*3*
	Chemo-based	74
	*TBF*	*38*
	*Bu Cy*	*1*
	*Bu Flu*	*3*
	*Flu Mel*	*2*
	*Treo-based*	*5*
	*Cy Flu*	*5*
	*Cy AraC Bu*	*19*
	*Cy Ida*	*1*
	RIC	*71* (*34%*)
	TBI based (*<6 Gy*)	*26*
	*Cy TBI*	*15*
	*Flu TBI*	*11*
	Chemo-based	*45*
	*TBF*	*8*
	*Bu Flu*	*4*
	*Bu ± others*	*1*
	*Flu Mel*	*6*
	*Bu Mel*	*1*
	*Treo-based*	*11*
	*Cy Flu*	*11*
	*Cy thiotepa Bu*	*1*
	*Cy AraC Bu*	*1*
GVHD prophylaxis	PT-Cy	118 (57%)
	ATG-based	90 (43%)

*Abbreviations*: *Tx* transplantation, *CR* complete remission, *ALL* acute lymphoblastic leukemia, *MRD* minimal residual disease, *Ph* Philadelphia, *CNS* central nervous system, *F* female, *M* male, *PB* peripheral blood, *BM* bone marrow, *D* donor, *R* recipient, *neg* negative, *pos* positive, *MAC* myeloablative conditioning, *TBI* total body irradiation, *Cy* cyclophosphamide, *Flu* fludarabine, *TBF* thiotepa, busulphan fludarabine, *Bu* busulphan, *Mel* melphalan, *Treo* treosulphan, *AraC* cytarabine, *Ida* idarubicine, *RIC* reduced intensity conditioning, *GVHD* graft-versus-host-disease, *PT-Cy* post transplant cyclophosphamide, *ATG* anti-thymocyte globulin

In Italics: details of cytogenetics ph negative patients and conditioning details

The median follow-up was 31 (range 2–79) months, and the median year of haplo-SCT was 2012 (range 2007–2014). Median age at haplo-SCT was 32 (range 18–76) years. The majority of patients (69%) had a Karnofsky performance status (KPS) ≥90%. Fourteen (7%) patients received a previous autologous SCT.

For patients with available information (*n* = 151), 100 (66%) patients had B ALL, and 51 (34%) T ALL.

Disease status at transplantation was CR1 in 91 (44%) patients, second or more complete remission (CR2+) in 58 (28%) and 59 (28%) patients were in active disease.

Cytogenetic analysis was available for 142 patients: 46 (32%) were Philadelphia positive and 96 (68%) were Philadelphia negative. Among Philadelphia negative patients, complete karyotype was reported for 69 patients. Thirty seven (54%) had a normal karyotype and 32 (46%) abnormal one. The most common alterations found were *t*(1;19) (*n* = 5), *t*(4;11) (*n* = 4), and *t*(12;21) (*n* = 2).

For patients with *t*(9;22), the use of tyrosine kinase inhibitors (TKI) was reported in 32 patients before transplant and in 11 patients after transplant, respectively.

Status of MRD at transplant for patients in CR was available for 91 of 149 patients, and 45 (49%) were MRD positive.

Conditioning regimen was MAC in 137 patients (66%) and RIC in 71 patients (34%), respectively. Among chemotherapy-based regimen TBF (thiotepa 10 mg/kg, fludarabine 150 mg/m^2^, busulfan 9.6 mg/kg i.v. for MAC; Thiotepa 5 mg/kg and busulfan 6.4 mg/kg i.v. for RIC) were the most commonly used regimens (Table 1).

GVHD prophylaxis was based on either PT-Cy in 118 (57%) or on ATG in 90 (43%) of the patients, respectively, in association with calcineurin inhibitors and mycophenolate mofetil (patient characteristics according to GVHD prophylaxis were shown in Additional file 1: Table S1). Among patients receiving an ATG-based GVHD prophylaxis details on the type of ATG was available for 66 patients (27 received Thymoglobulin and 44 Fresenius). According to the type of ATG, the median dose of ATG was 10 mg/Kg (total dose) for the thymoglobulin and 30 mg/Kg (total dose) for the Fresenius.

Stem cell source was peripheral blood (PB) or bone marrow (BM) in 119 (57%) and 89 (43%) patients, respectively, (Table 1).

### Engraftment and graft-versus-host-disease

**Fig. 1:**
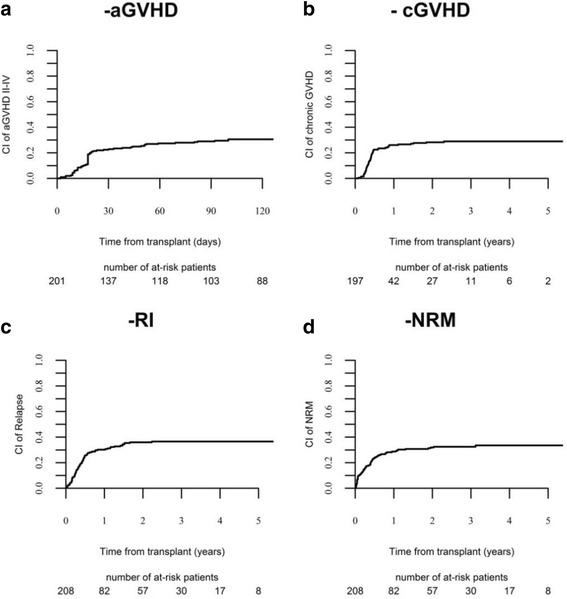
**a** aGVHD II–IV. **b** cGVHD. **c** relapse. **d** NRM after haplo-SCT for adults with ALL

Neutrophil engraftment was achieved in 92% of the patients. Median time to engraftment was 17 (range, 5–47) days. Seventeen patients (8.5%) experienced graft failure. The 100-day cumulative incidence (CI) of grade II–IV aGvHD was 30.6% (95% CI 24.3–37) (Fig. 1a) and 11% (95% CI 7.1–15.8) for grades III–IV, respectively.

In univariate analysis, the 100-day CI of grade II–IV aGvHD was different according to the stem cell source, being 17.3% (95% CI 10.2–26) for BM vs 40.8% (95% CI 31.6–49.7) for PB, grafts (*p* < 0.01).

Similarly, in the multivariate analysis (Table 2), the use of PB was significantly associated with an increased risk of aGVHD (HR 3.00, 95% CI 1.35–6.07, *p* < 0.01) compared to BM (Additional file 2: Figure S1). RIC conditioning was associated with a reduced risk of aGVHD as well (HR 0.46, 95% CI 0.23–0.93, *p* = 0.03).

**Table 2:** Multivariate analysis for outcomes

	HR	CI	*p*
	Relapse		
Age (per 10 years)	0.95	0.744–1.217	0.69
*Status at Tx*			
CR1 (reference)	1		
CR2+	1.69	0.72–3.95	0.22
Advanced	8.04	3.76–17.19	*<0.01*
Ph+ vs Ph−	1.09	0.53–2.24	0.80
KPS ≥ 90%	2.02	0.95–4.29	0.06
Female recipient	0.59	0.29–1.20	0.14
Female D ≥ male R	0.39	0.18–0.84	*0.01*
RIC vs MAC	1.32	0.67–2.5	0.41
R CMV positive	1.11	0.45–2.75	0.81
ATG vs PT-Cy	1.15	0.63–2.13	0.63
PB vs BM	1.46	0.68–3.10	0.32
Center (frailty)			0.94
	NRM		
Age (per 10 years)	0.98	0.72–1.33	0.90
*Status at Tx*			
CR1 (reference)	1		
CR2+	2.15	0.92–5.02	0.07
Advanced	1.50	0.59–3.82	0.39
Ph+ vs Ph−	1.45	0.61–3.42	0.39
KPS ≥ 90%	0.23	0.10–0.52	*<0.01*
Female recipient	1.26	0.52–3.04	0.59
Female D ≥ male R	1.47	0.60–3.57	0.39
RIC vs MAC	0.76	0.36–1.60	0.47
R CMV positive	0.57	0.23–1.41	0.22
ATG vs PT-Cy	1.79	0.90–3.55	0.09
PB vs BM	2.56	1.14–5.74	*0.02*
Center (frailty)			0.94
	LFS		
Age (per 10 years)	0.98	0.82–1.18	0.89
*Status at Tx*			
CR1 (reference)	1		
CR2+	1.91	1.06–3.41	*0.02*
Advanced	3.81	2.17–6.67	*<0.01*
Ph + vs Ph−	1.13	0.66–1.94	0.63
KPS ≥ 90%	0.78	0.47–1.29	0.34
Female recipient	0.77	0.45–1.32	0.35
Female D ≥ male R	0.72	0.41–1.24	0.24
RIC vs MAC	1.05	0.64–1.72	0.82
R CMV positive	0.73	0.40–1.33	0.31
ATG vs PT-Cy	1.32	0.84–2.06	0.21
PB vs BM	1.81	1.06–3.09	*0.02*
Center (frailty)			0.9
	OS		
Age (per 10 years)	1.02	0.84–1.22	0.82
*Status at Tx*			
CR1 (reference)	1		
CR2+	2.22	1.22–4.03	*<0.01*
Advanced	3.66	2.06–6.52	*<0.01*
Ph + vs Ph−	1.16	0.67–2.01	0.58
KPS ≥ 90%	0.62	0.37–1.02	0.06
Female recipient	0.81	0.47–1.38	0.44
Female D ≥ male R	0.76	0.43–1.35	0.36
RIC vs MAC	0.91	0.56–1.48	0.71
R CMV positive	0.81	0.43–1.51	0.51
ATG vs PT-Cy	1.4	0.88–2.20	0.14
PB vs BM	1.98	1.14–3.42	*0.01*
Center (frailty)			0.92
	GRFS		
Age (per 10 years)	0.91	0.76–1.08	0.29
*Status at Tx*			
CR1 (reference)	1		
CR2+	1.35	0.78–2.34	0.28
Advanced	2.71	1.56–4.68	**<** *0.01*
Ph + vs Ph−	1.20	0.72–2.02	0.47
KPS ≥ 90%	0.84	0.50–1.40	0.51
Female recipient	0.70	0.42–1.18	0.18
Female D ≥ male R	0.81	0.48–1.38	0.45
RIC vs MAC	1.03	0.65–1.63	0.87
R CMV positive	0.85	0.47–1.53	0.59
ATG vs PT-Cy	1.04	0.67–1.61	0.85
PB vs BM	1.82	1.10–3.01	*0.01*
Center (frailty)			0.92
	aGVHD II-IV		
Age (per 10 years)	0.75	0.56–1.00	0.05
*Status at Tx*			
CR1 (reference)	1		
CR2+	1.45	0.696–3.024	0.32
Advanced	0.76	0.29–2.0	0.59
Ph + vs Ph−	1.15	0.52–2.55	0.72
KPS ≥ 90%	0.80	0.33–1.93	0.63
Female recipient	1.23	0.53–2.88	0.62
Female D ≥ male R	1.84	0.82–4.13	0.13
RIC vs MAC	0.46	0.23–0.93	0.03
R CMV positive	1.28	0.43–3.78	0.65
ATG vs PT-Cy	0.97	0.52–1.81	0.93
PB vs BM	3.00	1.35–6.70	<0.01
Center (frailty)			0.93
	cGVHD		
Age (per 10 years)	0.97	0.69–1.37	0.89
*Status at Tx*			
CR1 (reference)	1		
CR2+	0.58	0.20–1.71	0.33
Advanced	0.62	0.20–1.90	0.40
Ph + vs Ph−	0.86	0.33–2.20	0.75
KPS 90%	0.68	0.26–1.77	0.43
Female recipient	0.93	0.34–2.57	0.90
Female D ≥ male R	1.38	0.48–3.94	0.54
RIC vs MAC	0.94	0.38–2.36	0.91
R CMV positive	0.51	0.16–1.56	0.24
ATG vs PT-Cy	0.67	0.25–1.73	0.41
PB vs BM	1.98	0.76–5.14	0.15
Center (frailty)			0.13

*Abbreviations*: *RI* relapse incidence, *NRM* non-relapse mortality, *LFS* leukemia-free survival, *OS* overall survival, *GRFS* refined graft-versus-host-free, relapse-free survival, *aGVHD* acute GVHD, *cGVHD* chronic GVHD, *Tx* transplantation, *CR* complete remission, *Ph* Philadelphia, *KPS* Karnofsky performance status, *D* donor, *R* recipient, *MAC* myeloablative conditioning, *RIC* reduced intensity conditioning, *ATG* anti-thymocyte globulin, *PT-Cy* post-transplant cyclophosphamide, *PB* peripheral blood, *BM* bone marrow

In Italics: details of cytogenetics ph negative patients and conditioning details

The 3-year CI of chronic GVHD was 29% (Fig. 1b), and CI of extensive cGVHD was 10%. No factors were significantly associated with cGVHD in the multivariate analysis (Table 2).

### Relapse incidence and non-relapse mortality

The 3-year CI of relapse was 37% (Fig. 1c), being 24% in patients in CR1, 32% for those in CR2+, and 60% in patients with advanced disease at transplantation (*p* < 0.01).

The impact of disease status remained significant in multivariate analysis (advanced HR 8.04, 95% CI 3.76–17.19, *p* < 0.01) and also the combination of female donor/male recipient was associated with a decreased risk of relapse in the multivariate analysis (HR 0.39, 95% CI 0.18–0.84, *p* = 0.01) (Table 2).

CI of NRM at 3 years was 32% (Fig. 1d). NRM was not influenced by disease status at haplo-SCT; it was 29% in CR1, 36% in CR2+, and 34% in patients with advanced disease, respectively, (*p* = 0.59). One hundred thirty-five patients died, 42 (31%) due to disease recurrence and 93 (69%) from NRM

Forty-eight (52%) patients died from infection, 24 (26%) from GVHD, 4 (5%) from hemorrhage, 1 (1%) from cardiac toxicity, 2 (2%) from sinusoidal obstruction syndrome (SOS), 3 (3%) from interstitial pneumonia, 6 (6%) from other transplant-related causes and 5 (5%) missing (Additional file 3: Table S2). In the multivariate analysis (Table 2), the risk of NRM was significantly lower in patients with a Karnofsky performance status ≥90% (HR 0.23, 95% CI 0.11–0.52, *p* < 0.01). The use of PB was associated with an increased risk of NRM (HR 2.56, 95% CI 1.14–5.74, *p* = 0.02).

### OS, LFS, and GRFS

With a median follow-up of 31 months, the probability of 3-year OS, LFS, and GRFS was 33, 31, and 26%, respectively.

**Fig. 2:**
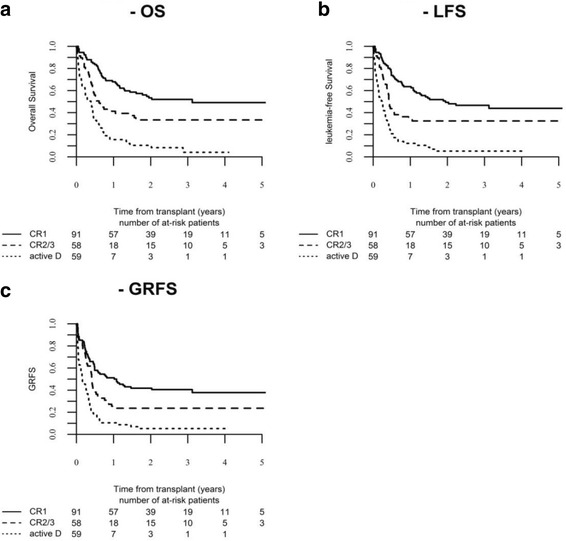
**a** OS. **b** LFS. **c** GRFS after haplo-SCT for adults with ALL according to disease status

OS, LFS, and GRFS were significantly different according to disease status: OS was 52% in CR1, 34% in CR2+, and 4% in advanced disease (*p* < 0.01); LFS was 47, 33, and 5 (*p* < 0.01); and GRFS was 41, 24, and 5 (*p* < 0.01), respectively, (Fig. 2a–c).

**Fig. 3:**
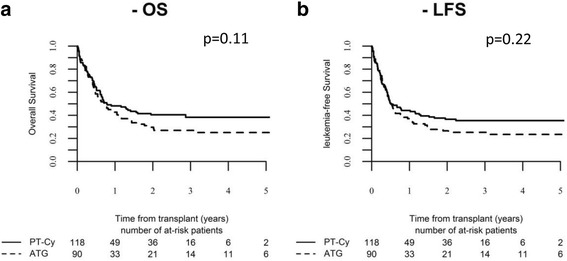
**a** OS. **b** LFS after haplo according to GVHD prophylaxis

Figure 3 shows OS and LFS according to the type of GVHD prophylaxis.

In the multivariate analysis (Table 2), disease status and the use of PB as stem cell source were found as negative prognostic factors for OS (CR2+ vs CR1: HR 2.22, 95% CI 1.22–4.03, *p* < 0.01; advanced vs CR1: HR 3.66, 95% CI 2.06–6.52, *p* < 0.01; PB vs BM: HR 1.98, 95% CI 1.14–3.42, *p* < 0.01), for LFS (CR2+ vs CR1: HR 1.91, 95% CI 1.06–3.41, *p* = 0.02; advanced vs CR1: HR 3.81, 95% CI 2.17–6.67, *p* < 0.01; PB vs BM: HR 1.81, 95% CI 1.06–3.09, *p* = 0.02) and for GRFS (advanced vs CR1: HR 2.71, 95% CI 1.56–4.68, *p* < 0.01; PB vs BM: HR 1.82, 95% CI 1.10–3.01, *p* = 0.01).

## Discussion

Allo-SCT remains the most effective post-remission treatment for adults with ALL and is the standard of care in high-risk patients including those with persistent or relapsing MRD, steroid and/or chemotherapy-resistance, and experiencing relapse after initial CR [9]. Several studies reported a 5-year LFS ranging from 40 to 60% in patients in CR1 receiving allogenic transplantation from an HLA-matched sibling or MUD [10–12]. Improvement of results over time has recently been reported [33].

Unfortunately, for a significant proportion of patients, an HLA identical donor cannot be identified leaving room for alternative approaches, such as transplantations from either mismatched unrelated or haploidentical donors. Thanks to easy access to haploidentical donors and to the introduction of innovative technologies, the procedure may be organized fast, avoiding delay caused by the search of unrelated donor. The attractiveness of haplo-SCT should be verified by detailed analysis of results, as potential advantages may be counterbalanced by increased risk of immune-related complications. As well, the impact on the incidence of relapse remains unknown and may vary according to diagnosis and disease status. Hence, there is need for studies focusing on homogenous populations in terms of the diagnosis. In the current one, the analysis was performed on adults with ALL, the area that has never been extensively explored.

In the largest report so far, Srour et al. [26] included 109 adults with ALL treated with haplo-SCT with GVHD prophylaxis uniformly based on PT-Cy. Only 32 patients were treated in CR1, and estimated 3-year LFS in this subgroup was 52%. The results of our study including 208 patients (44% in CR1) correspond well with the previous ones confirming the feasibility and efficacy of the procedure in this high-risk disease. However, in contrast to the study by Srour et al., we report results of two types of immunosuppressive regimen in the haplo-SCT setting. In our series, we did not find any difference in the outcome according to the type of GVHD prophylaxis except for a trend to increased risk of NRM in ATG group. This effect, however, did not reach statistical significance. Therefore, for adults with ALL in the haplo-SCT setting, both types of GVHD prophylaxis may be successfully used by transplant centers according to their policy.

As expected, disease status was the most important prognostic factor affecting relapse and survival. While the outcome of patients treated in CR1 appears comparable to results reported for HLA matched SCT in corresponding period [33], results of those with active disease remain very poor with only 5% OS reported at 3 years. Pavlu et al. recently reported results of HLA-matched SCT for patients with primary refractory ALL. The probability of LFS at 2 years was 28% [34]. However, in the setting of haplo-SCT results for patients with advanced disease status remain poor and efforts to achieve disease remission before transplant are needed. Modern approaches including bi-specific T cell engaging antibodies (blinatumomab) [35] or anti-CD22 immunoconjugates (inotuzumab ozogamycin) [36] have become available allowing a significant proportion of relapsed/refractory patients being bridged to SCT. As well, the use of chimeric antigen receptor (CAR) T cells [37, 38] is emerging as an effective approach for patients with lymphoid malignancies resistant to conventional chemotherapy.

In the study by Pavlu et al., [34] the outcome of patients with refractory disease was affected by the type of conditioning and donor/recipient-gender combination (better results for female donor to male recipient). In the current study, neither the type nor intensity of the conditioning had impact on outcome. This is consistent by previous findings by our group in the setting of unmanipulated haplo-SCT [39].

In our study, transplants from female donor to male recipient were associated with significantly reduced risk of relapse, without major impact on NRM and other outcomes. This observation suggests that ALL is particularly susceptible to graft-versus-leukemia reaction associated with mismatches of minor histocompatibility antigen encoded on Y chromosome.

Two retrospective studies comparing the type of stem cell source for haplo-SCT with PT-Cy were published showing no difference in terms of the incidence of GVHD and survival [40, 41]. Both analyses included RIC transplants and the populations with various myeloid and lymphoid malignancies. In contrast to the above-cited reports, the results of our study indicate a strong impact of the source of stem cells on outcome of unmanipulated haplo-SCT for adults with ALL. The use of PB was associated with significantly increased risk of acute GVHD and NRM, which translated into decreased survival, and leukemia-free survival. It must be stressed, however, that our population included both MAC and RIC procedures and different GVHD prophylaxis. The interaction between these variables and the effect of stem cell source on outcome should be considered and requires further exploration. With the rapid increase of unmanipulated haplo-SCT in the recent era the matter of optimal stem cell source needs to be addressed in larger homogenous population.

We are aware that in our study, there may be unmeasured factors that have not been considered, and this is a limitation when conducting retrospective studies. Some important data on cytogenetics and MRD are lacking, and also, the choice of the intensity and type of conditioning regimen, GVHD prophylaxis, and stem cell source are done according to each centers’ protocols and experience. Furthermore, the population of our study was homogenous in terms of the diagnosis, but ALL itself is a heterogeneous disease. In particular, treatment protocols differ for patients with Ph-positive and Ph-negative disease. Tyrosine kinase inhibitors are widely implemented in up-front treatment of Ph-positive ALL, but their use is also recommended in post-allo-SCT prophylaxis, which may influence the final outcome [42]. The retrospective nature of our study did not allow including this variable in the analysis. As well, data on minimal residual disease, being the most important prognostic factor in Ph-negative ALL, were unavailable [43].

## Conclusions

Despite above limitations, our results suggest that unmanipulated haplo-SCT is a valuable treatment option for adults with ALL with the great advantage of being able to quickly find a donor and avoid the risk of early relapse. Prospective studies are needed to compare the results of haplo-SCT with other types of donor on an intention-to-treat basis.

## Additional files

Additional file 1: Table S1. Patient characteristics according to GVHD prophylaxis. (DOCX 19 kb)

Additional file 2: Figure S1. Acute and chronic GVHD according to stem cell source. (DOCX 104 kb)

Additional file 3: Table S2. Causes of death according to GVHD prophylaxis. (DOCX 17 kb)

## Abbreviations

A: Acute
ALL: Acute lymphoblastic leukemia
allo-SCT: Allogenic hematopoietic stem cell transplantation
ATG: Anti-thymocyte globulin
BM: Bone marrow
Bu: Busulfan
c: Chronic
CAR: Chimeric antigen receptor
CI: Cumulative incidence
CR: Complete remission
DLIs: Donations or donor lymphocyte infusions
GRFS: GVHD-free, relapse-free-survival
GVHD: Graft-versus-host-disease
haplo-SCT: Haploidentical allo-SCT
KPS: Karnofsky performance status
LFS: Leukemia-free survival
MAC: Myeloablative conditioning
MRD: Minimal residual disease
MUD: Matched unrelated donor
NRM: Non-relapse mortality
OS: Overall survival
PB: Peripheral blood
PCR: Polymerase chain reaction
PT-Cy: Post-transplant cyclophosphamide
RI: Relapse incidence
RIC: Reduced intensity regimen
TBF: Thiotepa, busulphan, fludarabine
TBI: Total body irradiation
TCD: T cell depleted
TKI: Tyrosine kinase inhibitors
