# Supplementary material for: Unmanipulated haploidentical stem cell transplantation in adults with acute lymphoblastic leukemia: a study on behalf of the Acute Leukemia Working Party of the EBMT
Source: J Hematol Oncol. 2017 May 30;10:113. doi: 10.1186/s13045-017-0480-5 (PMC5450162; doi:10.1186/s13045-017-0480-5)
Supplement: Supplementary file 1 — Patient characteristics according to GVHD prophylaxis. (DOCX 19 kb) [file 13045_2017_480_MOESM1_ESM.docx]

| group | ATG | PT-CY | Test p-value |
| --- | --- | --- | --- |
| Number | 90 | 118 |  |
| **AGE median (range)** | 34(18-76.1) | 31.7(19-67.7) | 0.52 |
| **YEAR median (range)** | 2010(2007-2014) | 2013(2008-2014) | 0 |
| **disease status** |  |  |  |
| CR1 | 40 (44.4% ) | 51 (43.2% ) | 0.94 |
| CR2+ | 24 (26.7% ) | 34 (28.8% ) |  |
| active disease | 26 (28.9% ) | 33 (27.9% ) |  |
| **sex mismatch** |  |  |  |
| No F->M | 60 (66.7% ) | 84 (71.8% ) | 0.42 |
| F->M | 30 (33.3% ) | 33 (28.2% ) |  |
| missing | 0 | 1 |  |
| **Performance status** |  |  |  |
| KPS<90 | 23 (30.3% ) | 35 (31.8% ) | 0.82 |
| KPS≥90 | 53 (69.7% ) | 75 (68.2% ) |  |
| missing | 14 | 8 |  |
| **stem cell source** |  |  |  |
| BM | 26 (28.9% ) | 63 (53.4% ) | 0.001 |
| PB | 64 (71.1% ) | 55 (46.6% ) |  |
| **conditioning** |  |  |  |
| MAC | 58 (64.44% ) | 79 (66.9% ) | 0.70 |
| RIC | 32 (35.6% ) | 39 (33.1% ) |  |
| **conditioning details** |  |  |  |
| MAC TBI | 16 (17.8% ) | 47 (39.8% ) | 0.001 |
| MAC chemo | 42 (46.7% ) | 32 (27.1% ) |  |
| RIC TBI | 9 (10% ) | 17 (14.4% ) |  |
| RIC chemo | 23 (25.6% ) | 22 (18.6% ) |  |
| **acute GVHD** |  |  |  |
| no GVHD | 45 (50% ) | 52 (45.6% ) | 0.41 |
| aGVHD II-IV | 30 (33.7% ) | 31 (27.7% ) |  |
| **chronic GVHD** |  |  |  |
| No cGVHD | 66 (75% ) | 75 (68.8% ) | 0.33 |
| cGVHD | 22 (25% ) | 34 (31.2% ) |  |
| missing | 2 | 9 |  |
| **engraftment** |  |  |  |
| No | 6 (6.9% ) | 11 (9.6% ) | 0.48 |
| yes | 81 (93.1% ) | 103 (90.3% ) |  |
| missing | 3 | 4 |  |

Additional file 1: Table S1 Patients characteristics according to GVHD prophylaxis.
