# Supplementary figures and images for: Unmanipulated haploidentical stem cell transplantation in adults with acute lymphoblastic leukemia: a study on behalf of the Acute Leukemia Working Party of the EBMT
Source: J Hematol Oncol. 2017 May 30;10:113. doi: 10.1186/s13045-017-0480-5 (PMC5450162; doi:10.1186/s13045-017-0480-5)

Additional file 2: Figure S1 Acute and chronic GVHD according to stem cell source.
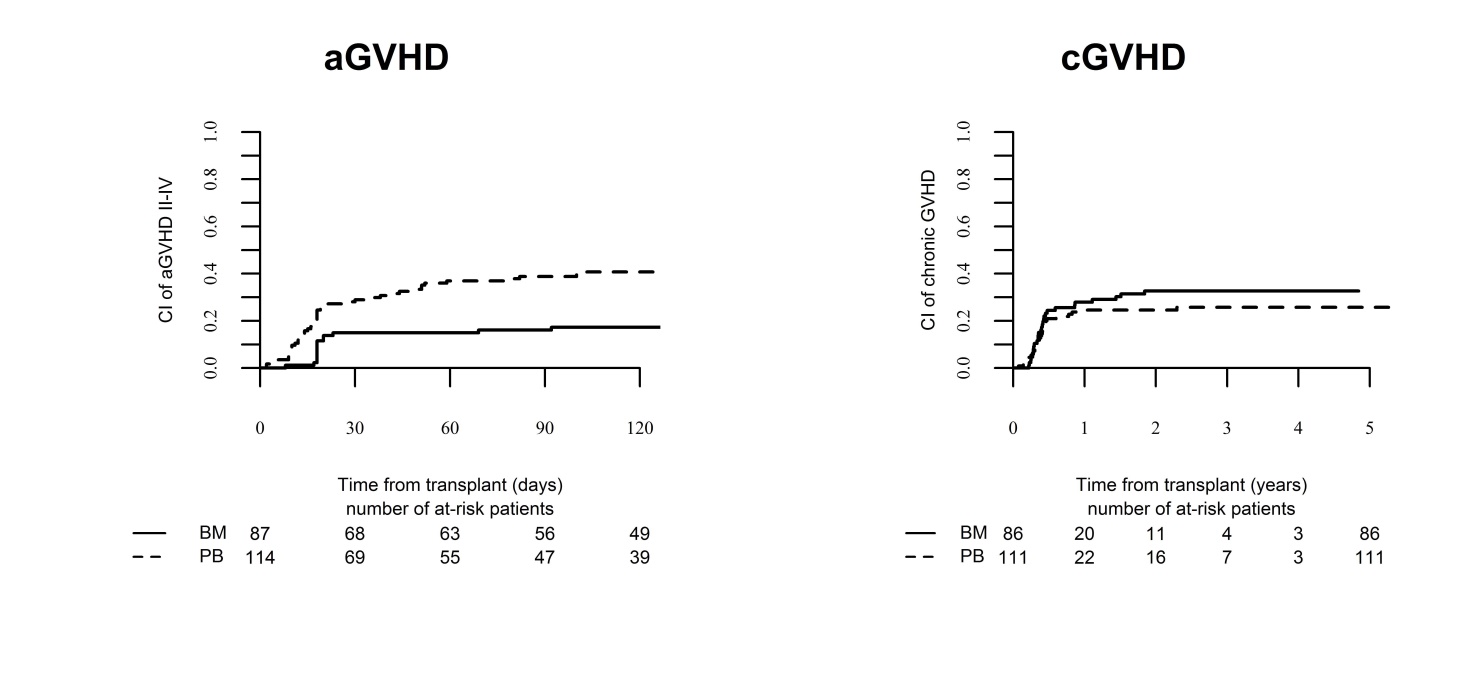


p=0.32

p<0.01

Supplement: Supplementary file 2 — Acute and chronic GVHD according to stem cell source. (DOCX 104 kb) [file 13045_2017_480_MOESM2_ESM.docx]
