# Supplementary material for: Unmanipulated haploidentical stem cell transplantation in adults with acute lymphoblastic leukemia: a study on behalf of the Acute Leukemia Working Party of the EBMT
Source: J Hematol Oncol. 2017 May 30;10:113. doi: 10.1186/s13045-017-0480-5 (PMC5450162; doi:10.1186/s13045-017-0480-5)
Supplement: Supplementary file 3 — Causes of death according to GVHD prophylaxis. (DOCX 17 kb) [file 13045_2017_480_MOESM3_ESM.docx]

| **causes of death** | ATG | PT-CY |
| --- | --- | --- |
| Number | 66 | 69 |
| Cardiac toxicity | 1 (1.59% ) | 0 (0% ) |
| Hemorrhage | 1 (1.59% ) | 3 (4.48% ) |
| VOD | 1 (1.59% ) | 1 (1.49% ) |
| Infection | 22 (34.92% ) | 26 (38.81% ) |
| Interstitial pneumonia | 2 (3.17% ) | 1 (1.49% ) |
| CGVHD | 15 (23.81% ) | 9 (13.43% ) |
| Original disease | 18 (28.57% ) | 24 (35.82% ) |
| other transplant related | 3 (4.76% ) | 3 (4.48% ) |
| missing | 3 | 2 |

Additional file 3: Table S2 Causes of death according to GVHD prophylaxis.
